# Targeted *Mybpc3* Knock-Out Mice with Cardiac Hypertrophy Exhibit Structural Mitral Valve Abnormalities

**DOI:** 10.3390/jcdd2020048

**Published:** 2015-04-21

**Authors:** Daniel P. Judge, Hany Neamatalla, Russell A. Norris, Robert A. Levine, Jonathan T. Butcher, Nicolas Vignier, Kevin H. Kang, Quangtung Nguyen, Patrick Bruneval, Marie-Cécile Perier, Emmanuel Messas, Xavier Jeunemaitre, Annemarieke de Vlaming, Roger Markwald, Lucie Carrier, Albert A. Hagège

**Affiliations:** 1 Faculté de Médecine, Université Paris Descartes, Sorbonne Paris Cité, Paris 75005 France; E-Mails: djudge@jhmi.edu (D.P.J.); patrick.bruneval@egp.aphp.fr (P.B.); emmanuel.messas@egp.aphp.fr (E.M.); xavier.jeunemaitre@inserm.fr (X.J.); 2 Division of Cardiology, Johns Hopkins University, Baltimore, MD 21287, USA; 3 Paris Cardiovascular Research Center, INSERM U970, Paris 75016 France; E-Mails: hnematalla@hotmail.com (H.N.); marie-cecile.perier@inserm.fr (M.-C.P.); 4 Department of Cardiology, Assistance Publique-Hôpitaux de Paris, Hôpital Européen Georges Pompidou, Paris 75016 France; E-Mail: quangtungdhyhn@yahoo.com (Q.N.); 5 Department of Regenerative Medicine and Cell Biology, Medical University of South Carolina, Charleston, SC 29403, USA; E-Mails: norrisra@musc.edu (R.A.N.); annemarieke.de.vlaming@gmail.com (A.V.); markwald@musc.edu (R.M.); 6 Cardiac Ultrasound Laboratory, Massachusetts General Hospital and Harvard Medical School, Boston, MA 02114 USA; E-Mail: rlevine@partners.org; 7 Department of Biomedical Engineering, Cornell University, Ithaca, NY 14853 USA; E-Mails: jtb47@cornell.edu (J.T.B.); hk576@cornell.edu (K.H.K.); 8 INSERM U582, University Pierre et Marie Curie-Paris 6, IFR14, Paris 75005 France; E-Mail: vigniernicolas@yahoo.fr; 9 Institut de recherche des Cordeliers, INSERM U652, Paris 75005, France; 10 Institute of Experimental and Clinical Pharmacology and Toxicology, University Medical Center Hamburg-Eppendorf, Hamburg 20246, Germany; E-Mail: l.carrier@uke.de

**Keywords:** mitral valve, TGFβ, hypertrophic cardiomyopathy

## Abstract

*MYBPC3* mutations cause hypertrophic cardiomyopathy, which is frequently associated with mitral valve (MV) pathology. We reasoned that increased MV size is caused by localized growth factors with paracrine effects. We used high-resolution echocardiography to compare *Mybpc3*-null, heterozygous, and wild-type mice (*n* = 84, aged 3–6 months) and micro-CT for MV volume (*n* = 6, age 6 months). *Mybpc3*-null mice showed left ventricular hypertrophy, dilation, and systolic dysfunction compared to heterozygous and wild-type mice, but no systolic anterior motion of the MV or left ventricular outflow obstruction. Compared to wild-type mice, echocardiographic anterior leaflet length (adjusted for left ventricular size) was greatest in *Mybpc3*-null mice (1.92 ± 0.08 *vs.* 1.72 ± 0.08 mm, *p* < 0.001), as was combined leaflet thickness (0.23 ± 0.04 *vs.* 0.15 ± 0.02 mm, *p* < 0.001). Micro-CT analyses of *Mybpc3*-null mice demonstrated increased MV volume (0.47 ± 0.06 *vs.* 0.15 ± 0.06 mm^3^, *p* = 0.018) and thickness (0.35 ± 0.04 *vs.* 0.12 ± 0.04 mm, *p* = 0.002), coincident with increased markers of TGFβ activity compared to heterozygous and wild-type littermates. Similarly, excised MV from a patient with *MYBPC3* mutation showed increased TGFβ activity. We conclude that MYBPC3 deficiency causes hypertrophic cardiomyopathy with increased MV leaflet length and thickness despite the absence of left ventricular outflow-tract obstruction, in parallel with increased TGFβ activity. MV changes in hypertrophic cardiomyopathy may be due to paracrine effects, which represent targets for therapeutic studies.

## 1. Introduction

Mitral valve (MV) abnormalities occur commonly in hypertrophic cardiomyopathy (HCM) [1]. The most frequent and predominant changes include elongation of the anterior (AML) and/or posterior (PML) mitral leaflets and anterior displacement of the MV and papillary muscles within the left ventricular cavity [2]. These changes and their severity contribute to both the initiation and magnitude of systolic anterior motion (SAM) of the MV with variable dynamic left ventricular outflow tract (LVOT) obstruction and, consequently, mitral regurgitation (MR) due to MV deformation with inadequate leaflet coaptation [3,4,5,6]. The etiology of MV leaflet elongation in this setting is not well understood, but it has long been attributed to turbulence in the LVOT leading to repeated deformation and stretch of the MV [7]. Recent investigation with 3-D echocardiography demonstrated that individuals with HCM and LVOT obstruction have larger MV leaflets than those with HCM without LVOT obstruction, yet both groups had greater MV area than normal controls [8]. If LVOT turbulence were required for MV enlargement, then we would not expect MV enlargement in people without a significant LVOT gradient.

Several other factors have been recognized as contributing to increased MV length and thickness in other disorders. In a murine model of Marfan syndrome with MV prolapse, fibrillin-1 deficiency results in diminished binding of the latent form of TGFβ and localized increase in this growth factor, accompanied by increased Ki67-positive cells in the MV [9]. Antagonism of TGFβ leads to normalization of MV length and thickness in that model [9]. X-linked MVP is caused by missense mutations in *FLNA*, the gene encoding filamin A [10]. Developmental studies in filamin-A-deficient mice show perturbation of matrix remodeling in AV valves [11]. Chromosomal loci have been recognized to associate with non-syndromic familial MV prolapse with prolongation and thickening of the valve leaflets [12,13,14]. In contrast with some of these conditions, MV disease in HCM is typically fibrotic rather than myxomatous, yet overlap among these disorders provides rationale for the hypothesis that they share common pathophysiology.

Loss-of-function mutations in *MYBPC3*, encoding cardiac myosin binding protein C (cMyBP-C), are a common cause of HCM in people [15,16]. Mice with *Mybpc3* knockout (−/−) develop concentric ventricular hypertrophy with histopathological features of HCM [17]. Importantly, these mice do not have SAM or LVOT obstruction in the setting of diminished systolic LV function. Although there are no reports of humans with homozygous null *MYBPC3* mutations, there are reports of people with severe LVH due to compound heterozygous truncating mutations in this gene [18,19]. We reasoned that investigation of the MV in this model of cMyBP-C deficiency could help to understand the pathogenesis of structural MV abnormalities in HCM. We hypothesized that increased growth factor production in or near the MV leaflets would arise from the *Mybpc3* mutation, and that MV pathology would occur in these mice in the absence of LVOT turbulence.

## 2. Experimental Section

Mice: The murine studies conform to the ARRIVE guidelines, and the protocol was approved by the Paris Cardiovascular Research Center’s Animal Care and Use Committee. Development of *Mybc3* (−/−) mice on a C57Bl/6 background strain was previously reported [17]. They were housed in a sterile facility at the Paris Cardiovascular Research Center using a standard diet.

Echocardiography: All echocardiograms were performed on adult mice aged 3–6 months, sedated with isofluorane using a VisualSonics Vevo 2100 imaging system and an MS400 (18–38 MHz) transducer (Fujifilm SonoSite, Inc.; Tokyo, Japan). The resolution for this device is 30 microns. The MV was imaged using a modified parasternal long axis view. Three separate measurements of the maximal diastolic length and thickness (at the mid-part of the leaflet) of AML and PML were obtained by a single observer blinded to the genotype of the mice on acquired cineloops using the zoom feature of the system, and a mean was calculated. Standard measures of the LV size, wall thickness, systolic LV function and left atrial anteroposterior dimension were included. We systematically searched for SAM using two-dimensional echocardiography and for LVOT flow acceleration using pulsed Doppler. Others have shown good correlation between this type of high frequency echocardiography and cardiac MRI in mice [20].

Micro-CT: Three consecutive mice of (−/−) and (+/+) genotype were chosen for high-resolution microCT imaging. MV annulus with intact leaflets and papillary muscle attachments were excised from mouse hearts after fixation in 10% buffered formalin. Cardiac muscle tissue was trimmed away from the annulus and chordal insertion sites prior to gross anatomical analysis and bright-field imaging. Leaflets were transferred to conical tubes containing 1% phosphotungstenic acid and imaged using Micro-CT as previously described [21]. Average leaflet thickness and volume and total leaflet area were measured using Microview (GE), an automated system that independently defines tissue boundaries, with a 25 µM^3^ resolution. 

Histology: Hearts were fixed in 10% buffered formalin, embedded in paraffin, and sectioned according to standard techniques [9]. Briefly, hearts were transected in the parasternal long axis to expose the MV and sectioned until MV leaflets were visible on unstained slides. Additional sections (10 μM thick, 4–6 sections) were mounted onto slides for additional analysis. To assess these MV leaflets for increased collagen, staining with modified Movat’s techniques was performed in three mice of each +/+ and −/− genotypes. Immunostaining for periostin was performed using a rabbit polyclonal antibody (Abcam #ab14041) in three mice of each +/+ and −/− genotypes. To assess for additional markers of TGFβ signaling, immunostaining for phosphorylated Smad2 (pSmad2) was performed using a rabbit polyclonal antibody (Millipore #AB3849, Merck KGaA, Darmstadt, Germany) in three mice of each +/+ and −/− genotypes. Standard chromogens (Diaminobenzidine (DAB) or 3-Amino-9-Ethylcarbazole (AEC)) were used according to manufacturer’s protocols (Vector Laboratories, Inc; Burlingame, CA; USA). All images were reviewed, and the amount of chromogen corresponding to target ligand was assessed by three blinded investigators. Relative quantification was determined by an arbitrary scale, ranging from (1) for no staining, to (5) for prominent staining; histology scores were averaged among the three observers. These investigations were used to support more quantitative methods of mRNA and protein analysis when differences were identified.

RNA analysis: In order to quantify differences observed with immunostaining, qRT-PCR expression assays were used to compare the mRNA expression of two genes that are induced by TGFβ: *Postn* encoding periostin and *Ctgf* encoding connective tissue growth factor (CTGF). The expression of these genes was compared in (+/+) and *Mybpc3* (−/−) MV, adjacent septum, and apex of the LV. For RNA extraction hearts were flushed with normal saline, and the MV was removed with a dissecting microscope, followed by immersion in Trizol (Invitrogen, Thermo Fisher Scientific, Waltham, MA, USA. Sections of adjacent (septum) and distant (apex) LV myocardium were also dissected and immersed in Trizol. RNA was extracted as previously reported [9]. Real-time PCR reactions were performed and analyzed using ABI PRISM 7900 (Applied Biosystems, Thermo Fisher Scientific, Waltham, MA, USA). Levels of mRNA for *Ctgf* and *Postn* were assessed in relation to expression of *Gapdh* using specific primer-probe from Applied Biosystems. Each experiment was performed in triplicate, blinded to MV imaging results, with three consecutive mice of each genotype at 6 months of age and with specimens from the same site in each genotype. 

Protein analysis: Murine MV leaflets were dissected and snap frozen in lysis buffer. After homogenization, protein was quantified and equal amounts (4 μg) separated by gel electrophoresis as previously described [22]. The periostin primary antibody for Western analysis was generously provided by S. Hoffman, Medical University of South Carolina, Charleston, SC, USA. Periostin was normalized to β-actin to account for differences in protein loading. 

Complementary human studies: Written informed consent was obtained from the individual who provided MV tissue. The study was conducted in accordance with the Declaration of Helsinki, and the protocol was approved by the Ethics Committee (CPP Ile de France VI, 25 June 2008) and Biomedicine Agency for genetic studies approval (12 December 2008). Movat’s staining and immunohistochemical analyses were performed per standard protocols and as previously described (20). Proteins were extracted from excised MV leaflets of a 71-year-old patient with non-obstructive HCM due to *MYBPC3* mutation who underwent mitral valve surgery for chordal rupture and from normal control valves (*n* = 3, obtained from normal hearts that were not used for cardiac transplant) that had been previously processed, sectioned, and mounted. Valve sections were photographed and area measurements were used to equalize tissue amounts. Proteins were de-crosslinked and extracted using the Qproteome FFPE Tissue Extraction Kit according to manufacturer’s recommendations (Qiagen, Hilden, Germany). Extracted protein lysate was mixed with appropriate amounts of 2 × SDS page loading dye and run on denaturing SDS-PAGE. Blots were probed with an α-Collagen-I antibody (1:1000, MD Biosciences GmbH, Zürich, Switzerland) and a secondary HRP-labeled goat-anti-rabbit antibody (Sigma-Aldrich, St. Louis, MO, USA) (1:7500). Bands were detected with Super Signal West Femto Substrate (Thermo Fisher Scientific, Waltham, MA, USA) and densitometric analyses were performed using Adobe Photoshop CS5. Analyses were performed in triplicate and results are graphically depicted. 

Statistics: Statistical analysis for the MV comparisons used the SAS version 9.2 (statistical analysis system, Cary, NC, USA). MV leaflet length, LV end-diastolic volume and LV mass reached statistical criteria for normal distribution across groups, allowing the use of analysis of variance for comparisons. Comparisons of variables across groups were performed using analysis of variance without and with adjustment for LV mass and LV end-diastolic volume. A two-tailed *p* value <0.05 was considered as statistically significant. Pairwise comparisons were also analyzed using analysis of variance and p-value, adjusted for multiple testing (Tukey adjustment). Intra-observer and inter-observer variability of ultrasonic measurements was assessed blindly by linear regression analysis (*R*^2^) using five separate measures in randomly chosen mice of the three genotypes (*N* = 15 for each). Results are provided with mean ± standard deviation, range.

## 3. Results and Discussion

### 3.1. Mybpc3 (−/−) Anterior Mitral Leaflet is Longer and Thicker than Wild-Type Anterior Mitral Leaflet by Echocardiographic Analysis

**Figure 1:**
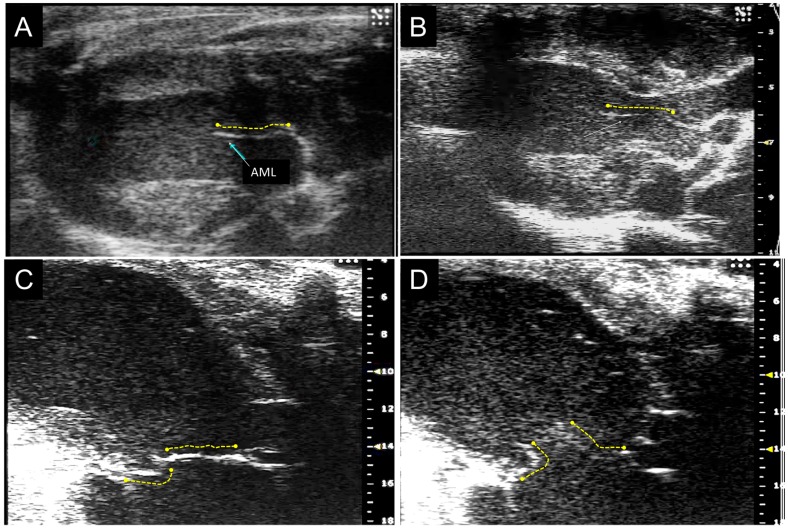
Imaging of the MV by high-resolution echocardiography. Representative echocardiographic images are shown from *Mybpc3* (−/−) (**A**,**C**,**D**) and (+/+); (**B**) mice; arrows show AML; Systolic (**C**) and diastolic (**D**) images are shown with AML and PML highlighted.

Data acquired by echocardiography are summarized in Table 1. The *Mybpc3* (−/−) mice had LV globular dilation with increased wall thickness and mass compared to both wild-type (+/+) and heterozygous (+/−) mice as previously reported [17]; neither SAM nor LVOT flow acceleration was present. The length of the anterior MV leaflet was greater in the (−/−) mice than in the (+/−) and (+/+) mice (1.92 ± 0.08 *vs.* 1.73 ± 0.07 *vs.* 1.73 ± 0.08 mm, respectively; *p* < 0.0001). After correction for LV mass, as well as for LV end-diastolic volume (EDV), or both, these differences remained highly significant (*p* < 0.001) (Figure 1, Table 1). The length of the posterior MV leaflet was similar in all three groups of mice. The aggregate MV leaflet thickness (AML + PML thickness) in (−/−) was greater than in (+/+) mice (0.23 ± 0.04 *vs.* 0.15 ± 0.02 mm, respectively; *p* < 0.001). Analysis of reproducibility of the echocardiographic measurements showed small intra-observer and inter-observer variability, with calculated *R*^2^ of 0.88 and 0.90, respectively.

### 3.2. MicroCT Imaging Confirms the Results of Echocardiographic Imaging

The MV volume by microCT was greater in (−/−) mice than (+/+) mice (0.47 ± 0.06 *vs.* 0.15 ± 0.06 mm^3^, respectively; *p* = 0.018). Similarly, the MV thickness in (−/−) mice was greater than in (+/+) mice (0.35 ± 0.02 *vs.* 0.12 ± 0.02 mm, respectively; *p* = 0.002). Total leaflet surface area was 7.35 ± 0.84 mm^2^ in (+/+) mice *vs.* 3.68 ± 1.16 mm^2^ in (+/+) mice (*p* = 0.06).

**Table 1:** Cardiac findings using high resolution echocardiography.

Parameter	+/+	+/−	−/−	*p*
	N = 22	N = 33	N = 29	
**Length AML (mm)**	1.72 ± 0.08	1.73 ± 0.07	1.92 ± 0.08	<0.001 *
	(1.64–1.94)	(1.55–1.90)	(1.80–2.09)	
**Length PML (mm)**	1.36 ± 0.07	1.33 ± 0.07	1.35 ± 0.07	NS *
	(1.23–1.46)	(1.20–1.45)	(1.23–1.51)	
**Thickness AML + PML (mm)**	0.15 ± 0.02	0.18 ± 0.04	0.23 ± 0.04	<0.001 *
	(0.11–0.18)	(0.10–0.19)	(0.14–0.29)	
**LV Mass (mg)**	80 ± 16	88 ± 7	129 ± 26	<0.0001
	(55–109)	(71–105)	(99–173)	
**EF (%)**	63 ± 5	62 ± 7	35 ± 7	<0.0001
	(55–76)	(52–80)	(22–49)	
**ED Volume (mL)**	67 ± 11	63 ± 11	118 ± 40	<0.0001
	(43–88)	(39–89)	(42–175)	

(*) indicates *p* values that are adjusted for LV mass and end-diastolic volume. Results are expressed as mean ± SD (range). All mice are age 3–6 months.

### 3.3. *Mybpc3*-(−/−) Mitral Valve Leaflets Have Evidence of Increased TGFβ Signaling

**Figure 2:**
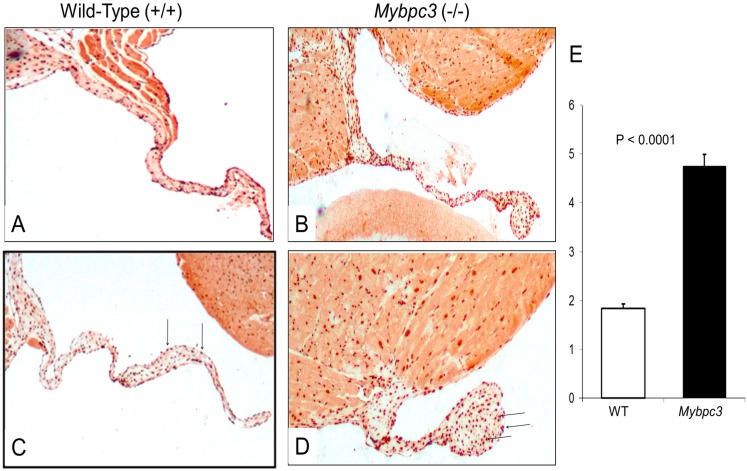
*Mybpc3* (−/−) MVs have increased nuclear pSmad2. Immunostaining for pSmad2 was performed using AEC as the chromogen (red) according to standard protocols (Vectorlabs). Representative 40× images are shown from (+/+) (**A**,**C**) and *Mybpc3* (−/−) (**B**,**D**) mice. Nuclear pSmad2 staining (arrows) is a marker for TGFβ activity. Relative quantification of immunostaining (**E**) was performed by blinded reviewers, using a scale from 1 (faint staining) to 5 (strong staining). Error bars show SEM.

TGFβ is a growth factor that has been directly implicated in HCM pathogenesis, and its activity is assessed by downstream effects [23]. The *Mybpc3* (−/−) MV leaflets have increased nuclear pSmad2 immunostaining (Figure 2). Smad2 is a receptor-activated intracellular protein that becomes phosphorylated in response to TGFβ ligand interacting with the TGFβ receptor [24]. Upon phosphorylation, it combines with Smad4 and translocates to the nucleus. Nuclear immunostaining of pSmad2 is a commonly used marker of canonical TGFβ stimulation [25].

**Figure 3:**
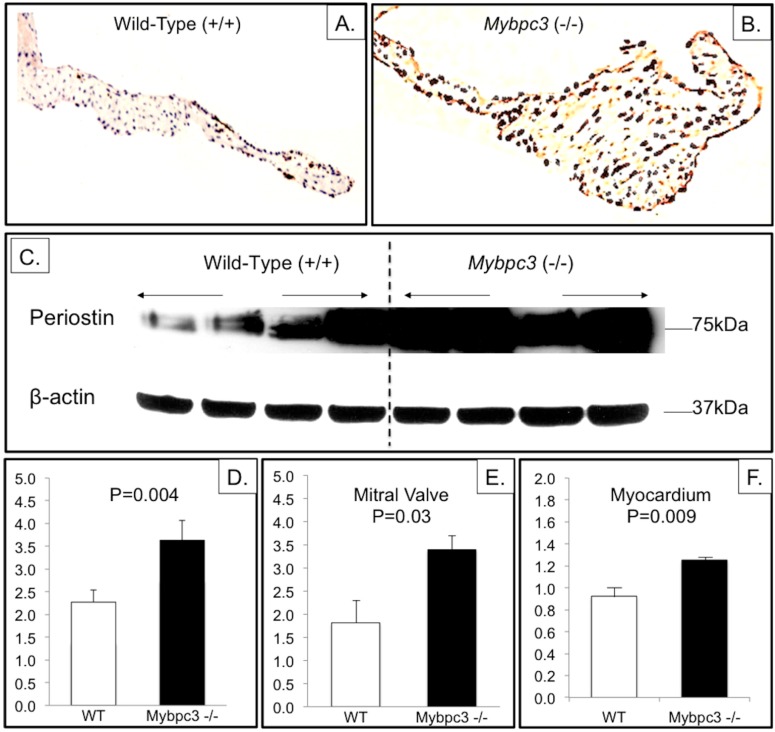
*Mybpc3* (−/−) MVs have increased periostin. Immunostaining for periostin was performed using DAB as the chromogen (brown). Representative low power (40×, **A**,**B**) images are shown from (+/+) (**A**) and *Mybpc3* (−/−) (**B**) mice. Increased periostin (brown) is evident in the *Mybpc3* (−/−) MV leaflets; (**C**) Western blot for quantification of periostin in MV leaflets comparing 4 (+/+) and 4 (−/−) leaflets, with use of β-actin to normalize for protein loading. Relative quantification of immunostaining (**D**) was performed by blinded reviewers using a scale from 1 (faint staining) to 5 (strong staining). Quantification of Western blots was performed by NIH ImageJ software, demonstrating increased periostin in both MV leaflets (**E**) and adjacent myocardium (**F**) in *Mybpc3* (−/−) mice compared to wild-type controls (+/+). Error bars show SEM.

Periostin is a secreted protein that is induced by TGFβ and regulates collagen production [22]. To investigate a downstream marker of TGFβ signaling, we analyzed periostin in both (−/−) and (+/+) mice. We reasoned that increased periostin could contribute to the MV phenotype in *Mybpc3* (−/−) mice. Indeed, immunostaining for periostin was markedly increased in the *Mybpc3* (−/−) mice compared to (+/+) mice (Figure 3). In order to quantify the increase in periostin in the *Mybpc3* (−/−) mouse MV and myocardium, we performed Western analyses (Figure 3). The ratio of periostin to β-actin in MV leaflets was greater in the (−/−) than in the (+/+) mice (3.40 ± 0.29 *vs.* 1.81 ± 0.50, respectively; *p* = 0.034). The ratio of periostin to β-actin in the myocardium was also greater in the (−/−) than the (+/+) mice (1.25 ± 0.01 *vs.* 0.92 ± 0.09, respectively; *p* = 0.009).

**Figure 4:**
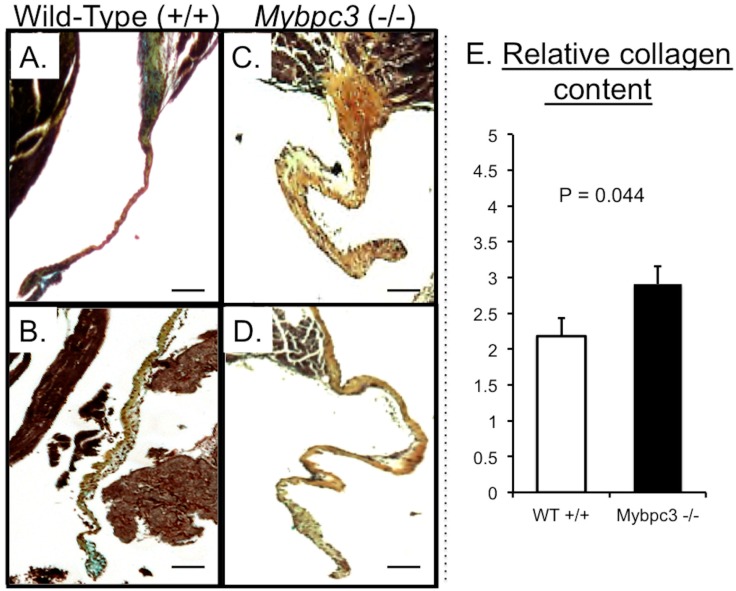
*Mybpc3* (−/−) MVs have more collagen. MVs were stained with modified Movat’s (**A**–**D**). The *Mybpc3* (−/−) MV leaflets (**C**,**D**) show more collagen (yellow−brown in **C** and **D**) than the Wild-Type (+/+) MV leaflets (**A**,**B**). All size bars correspond to 100 μM. Relative quantification of collagen (**E**) on Movat’s staining was performed by blinded reviewers, using a scale from 1 (blue valves without brown) to 5 (brown valves without blue). Error bars show SEM.

Increased TGFβ activity stimulates production of collagen in MV leaflets in mice as shown using Movat’s stains (Figure 4). The mouse MV leaflets in (−/−) mice have a greater abundance of collagen than in (+/+) mice, as determined by scoring for brown shading by blinded review of Movat’s staining (Figure 4). 

### 3.4. Quantitative PCR Analysis Confirms Increased Expression of TGFβ-Related Genes in Myocardium

**Figure 5:**
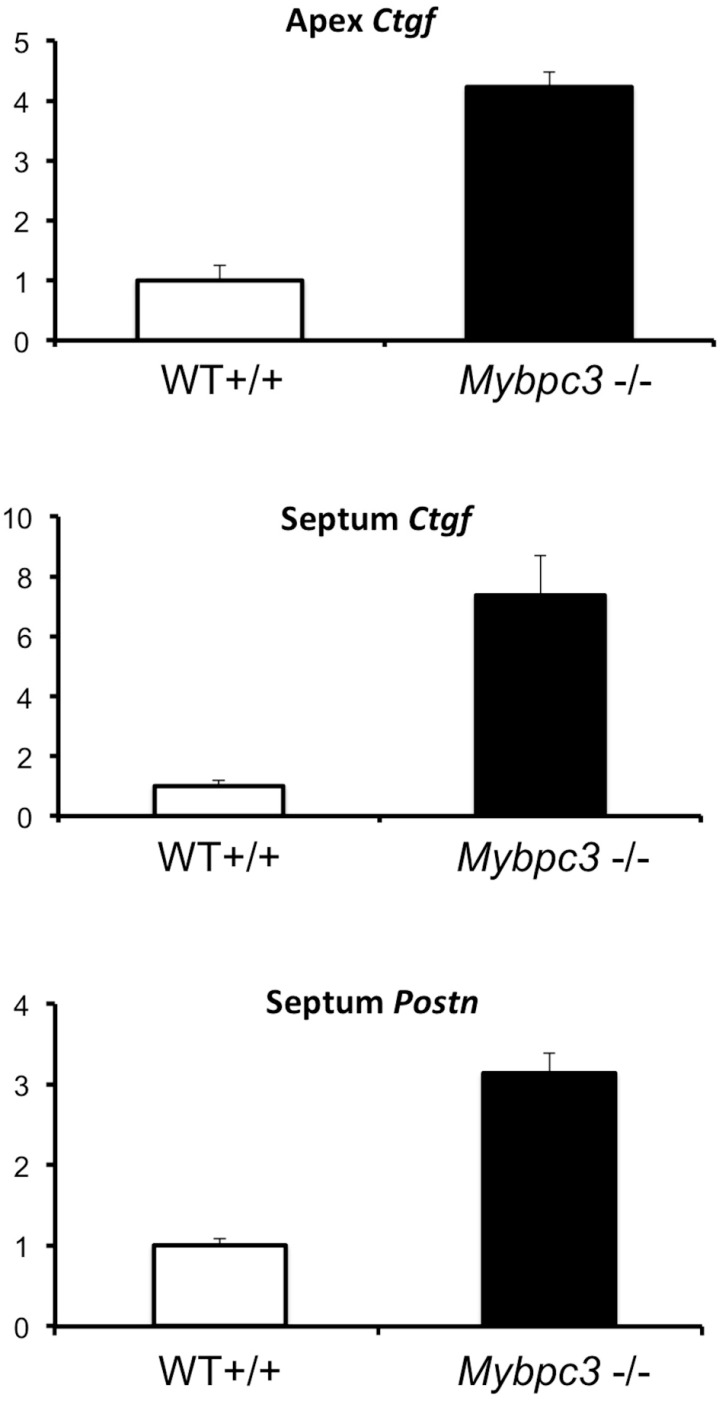
Increased expression of *Ctgf* and *Postn* in *Mybpc3* (−/−) hearts. The relative mRNA expression of *Ctgf*, encoding connective tissue growth factor, and *Postn*, encoding periostin, is shown with the wild-type (+/+) level set at 1.0. Bar graphs show the average relative gene quantification (N = 3 for each genotype and each tissue) with SEM.

Connective tissue growth factor is also produced in response to TGFβ, and increased expression of *Ctgf* mRNA has been demonstrated in another murine model with cardiac hypertrophy due to a sarcomere mutation [23]. Both the septum and apex from the *Mybpc3* (−/−) mice had markedly increased expression of *Ctgf* compared to (+/+) mice (4.2-fold increase for apex and 7.1-fold increase for septum, *p* = 0.0008 and 0.004 respectively) (Figure 5). The septum also had increased expression of *Postn* in the *Mybpc3* (−/−) compared to (+/+) mice (3.1-fold increase, *p* = 0.01), but there was similar expression of *Postn* at the apex (*p* = 0.6). The expression of genes encoding these two growth factors was similar when comparing RNA derived from the MV in the *Mybpc3* (−/−) and (+/+) mice (*p* = 0.3 for *Ctgf* and 0.5 for *Postn*) suggesting that the primary source for the increased protein present in MV leaflets is due to paracrine effects mediated by myocardial hypertrophy in adjacent myocardium.

### 3.5. MV Leaflets in Human Non-Obstructive Hypertrophic Cardiomyopathy (HCM) Exhibit Increase in Collagen along with Molecular Changes in TGFβ Signaling

**Figure 6:**
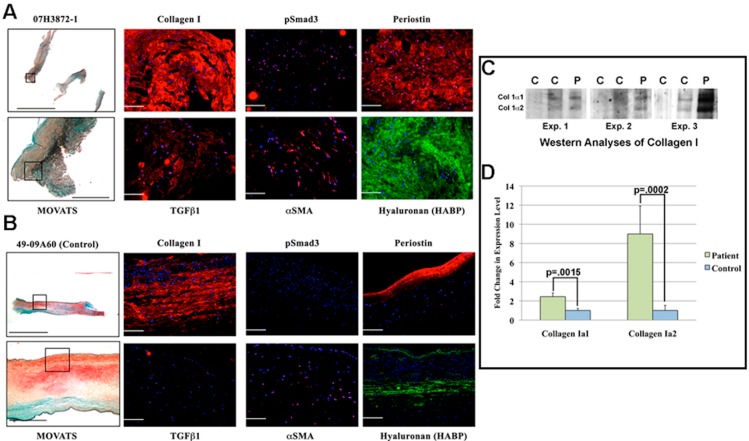
Comparison of excised MVs in an HCM patient with a *MYBPC3* mutation and in two normal hearts (controls, *n* = 2). Findings in the patient (**A**) and in a representative control (**B**) with histological (Movat’s) and immunohistochemical stainings are shown. Boxed areas represent regions where protein stainings were captured. (**A**) The MV from the HCM patient exhibits a disruption of the normal stratified layers of the mitral leaflets with an increase in TGFβ downstream extracellular matrix proteins (collagen I, periostin, and hyaluronan). Higher levels of TGFβ1 were observed in the leaflets of the HCM valve as well as the TGFβ1 signaling intermediate, pSmad3. Moreover, α-SMA, a TGFβ-regulated gene and a marker of myofibroblast phenotype, was also increased in these leaflets. All proteins are depicted in red except hyaluronan, which is in green, while nuclei are blue; (**C**) Western analysis of excised proteins collected from the valve revealed a significant increase in both collagen 1α1 and collagen 1α2 in the HCM patient with *MYBPC3* mutation compared to the controls. Shown are each of the three Westerns performed on three different areas of the same human valve on the de-crosslinked proteins from human valve sections; they were compared against comparable areas in three separate controls (two controls are shown on the Westerns). Sample size was normalized based on similar tissue volumes used in the valve extraction procedure (see Experimental Section) (C = controls, P = patient); (**D**) Graphical depiction of fold-change in collagen expression (*N* = 3).

These data were obtained to show that similar MV changes do in fact occur in the human HCM model with *MYBPC3* mutation, with histopathologic changes corresponding to the elongation described clinically by echo and anatomic pathology. It is unusual to obtain such a specimen, since these valves are only rarely excised. Therefore, only one MV specimen is available from an individual with HCM and *MYBPC3* mutation (p.Arg326Gln), which has previously been reported to cause HCM [26,27]. It was compared with normal human MVs obtained from unused normal cardiac transplant donor controls. Formalin-fixed, paraffin-embedded (FFPE) sections from an individual with *MYBPC3* mutation who underwent MV surgery were analyzed for TGFβ effectors. Immunofluorescence studies demonstrate an increase in the TGFβ-responsive extracellular matrix genes (collagen I, periostin, and hyaluronan), as well as an increase in both the TGFβ1 ligand and TGFβ1 signaling intermediate pSmad3 (Figure 6). Because TGFβ1 has previously been demonstrated as promoting a myofibroblastic phenotype during valve disease pathogenesis, we additionally examined the expression of α-smooth muscle actin (α-SMA). As shown in Figure 6, there was an increase in α-SMA expressing cells in the MV with *MYBPC3* mutation compared to controls. In order to quantify the increased level of type 1 collagen in *MYBPC3*-mutant human MV leaflets, equal tissue volumes were analyzed by Western analysis. Proteins were de-crosslinked and isolated from deparaffinized FFPE MV tissue sections. Compared to control MV tissues, the HCM-associated MV leaflets had a >2-fold increase in collagen type I, αI (COL1A1) and a >-9-fold increase in collagen type I, α2 (COL1A2; p.0015 and .0002, respectively) (Figure 6). This demonstrates that similar MV abnormalities occurred in this person with HCM, although it is a single example. As such, it is not possible to draw broader conclusions from this individual.

### 3.6. Discussion

Our results demonstrate that MV elongation and thickening occur in cMyBPC-null mice despite the absence of SAM or obstruction, and that the magnitude of excess tissue is independent of LV enlargement and hypertrophy. After adjustment for LV mass and size, high-resolution echocardiography shows a consistent 12% greater length in AML for the *Mybpc3* (−/−) mice, while the more detailed three-dimensional micro-CT measurements in a subset show 3-fold greater volume in the (−/−) as compared to (+/+) mice. Immunohistochemical analysis of the MV and adjacent LV myocardium in knock-out mice demonstrates increased collagen, which was also demonstrated in excised MV leaflets from an HCM patient with a *MYBPC3* mutation. Increased expression of growth factors in the LV myocardium of *Mybpc3* (−/−) strain of mice correlates with MV remodeling, which is consistent with the proposal that these growth factors contribute to its pathogenesis.

The mRNA expression of the genes encoding both periostin and CTGF was similar in the MV leaflets themselves, yet greater in the adjacent myocardium. The differences in periostin gene expression are greatest in the nearby myocardium, in contrast with the more homogeneously increased expression of CTGF. Within the MV leaflets, there is no significant difference in expression of these genes at this age (6 months), yet histological and quantitative protein (Western) analyses show increased periostin in the MV leaflets. This indicates a paracrine effect, wherein the origin of the increased protein is the adjacent myocardium, and its target is the nearby mitral valve. 

Elongation of the MV leaflets commonly occurs in HCM [3,4]. A landmark study of MV abnormalities in HCM was published in 1992 [28]. In that report, the average leaflet area of the excised MV among a cohort of 94 patients with obstructive HCM was 12.9 ± 3.7 cm^2^, as compared with a cohort of 45 normal controls wherein the MV leaflet area was 8.7 + 2.0 cm^2^; *p* < 0.001). Notably, the MV surface area in those with HCM widely varied, ranging from 6.3 to 23.0 cm^2^, and 55 of the 94 (59%) had a MV surface area >95 percentile of the normal control MVs [28]. Another group found a wide spectrum of MV leaflet abnormalities among individuals undergoing surgical intervention for HCM with SAM, including degenerative, myxomatous, restrictive, and elongated AML as well as restrictive chordae and abnormal position of the papillary muscles [29]. This variability may reflect the intrinsic heterogeneity for the genetic causes in HCM, as well as the variability in other genetic and environmental factors that normally occur in patients. Our investigation utilized an inbred strain of mice in order to circumvent these factors.

The specificity of MV enlargement in HCM *vs.* other causes of LV hypertrophy was addressed in at least one prior pathological report in which sizes of excised MVs were compared among patients with aortic valve stenosis or regurgitation, with or without LV dilation, to MV size in HCM and controls [30]. In this study, compared to controls, MV size was similarly increased in HCM and in aortic stenosis patients with LV dilation, but remained unchanged when aortic stenosis occurred without LV dilation despite LVH.

There are many severe consequences of MV leaflet elongation in HCM [1,6,31]. First, elongation of the AML may result in displacement of a portion of the excess tissue interposed into the pathway of the LVOT at the beginning of systolic ejection, thereby facilitating SAM and obstruction and causing MR, the severity of which increases proportionally to the degree of mismatch between the AML and PML length [32]; it also worsens left atrial enlargement and increases the risk for atrial fibrillation. Finally, obstruction increases the likelihood of death and progressive heart failure [33].

Although leaflet elongation in HCM has long been attributed to MV deformation with repeated stretch of the MV due to SAM, enlarged MV leaflets have also been reported in the absence of LVOT obstruction [8,28]. Two studies have recognized that healthy carriers of a gene mutation predisposing to HCM (without LVH) also have pathologic elongation of the AML [3,4]. This suggests that a process in the surrounding pre-hypertrophic LV exerts an early effect on the MV leaflets. Since the sarcomere proteins in which mutations typically occur in HCM are not abundant in the MV leaflets, it supports our hypothesis that increased expression of paracrine growth factors may arise in the surrounding myocardium where sarcomere proteins are abundant, with influence on the size of the adjacent MV. Our results in this murine model and in 1 person with HCM with a *MYBPC3* mutation confirm and extend this hypothesis, with recognition of specific alterations in the TGFβ pathway that contribute to both LVH and MV disease in this setting. While there may be a role for LVOT obstruction contributing to MV pathology, better models of HCM with and without obstruction are needed to address this issue.

Several lines of evidence emphasize the dynamic nature of MV disease in different contexts. MV area is increased by approximately 30% in people with inferior wall myocardial infarction and dilated cardiomyopathy compared to people with normal hearts [34]. A study in sheep that compared MV remodeling in response to inferior myocardial infarction with or without LV patch to prevent LV dilation showed that the increase in MV area correlates with LV dilation [35]. Thus, while it cannot be excluded that LV dilatation might have contributed to leaflet elongation in *Mybpc3*-mutant mice, the LV enlargement cannot solely explain the difference. Moreover, the MV leaflets do not appear tethered (Figure 1) or drawn down apically into the ventricular cavity with apically restricted closure and leaflet tenting, as sometimes occurs in dilated or ischemic cardiomyopathy [34,35]. Based on the absence of tethering or leaflet tenting, LV enlargement is unlikely to be the sole explanation for the leaflet enlargement. Our data suggest that increased growth factor signaling contributes to MV enlargement. It seems that increased MV area in dilated or ischemic cardiomyopathy is a limited process that is insufficient for preventing the occurrence of MR, while in HCM, greater proportional increases in MV size are observed, as shown here and in human studies. 

Mice with a knock-in mutation in *Fbn1*, the gene encoding fibrillin-1, develop many systemic features of Marfan syndrome [9,36,37]. These mice similarly have pathological elongation and thickening of the MV leaflets [9,38]. Fibrillin-1 binds the inactive form of latent TGFβ, and its deficiency has been shown to result in increased activation and signaling of this pluripotent growth factor, including in the MV leaflets [9]. Therapies that modulate excessive TGFβ stimulation of MV leaflets have been shown to normalize the MV in *Fbn1*-mutant mice [9]. 

HCM is typically caused by dominant mutations in genes encoding elements of the cardiac sarcomere [39]. The process whereby sarcomere gene mutations result in HCM has been elegantly investigated over the past two decades. Recent work in this area has led to improved understanding of the role of a cascade of gene expression in non-myocyte cells within the LV that results in proliferation of non-myocyte cells and increased fibrosis [23]. Periostin contributes to this process, but *Postn*-null mice with an α-myosin heavy chain gene mutation and LVH are only partially normalized compared to those with normal periostin [23]. A critical role for TGFβ signaling in this process was demonstrated by gene expression analysis, immunostaining, and TGFβ-neutralizing therapies [23]. Our work extends these findings to the MV in HCM, providing both an explanation for MV disease in this disorder and an opportunity to investigate treatment for this with TGFβ-neutralizing therapies. Although this strain of mice does not have LVOT turbulence, it does have LVH due to a sarcomere gene mutation, and thus only partly recapitulates HCM.

One potential limitation in our analysis is the issue of blinding. Although the valve measures were made in a blinded fashion, with magnification to limit the ability of the interpreter to see the LV size and function, it is possible that bias could have entered into the analysis of echocardiograms. Thus, the microCT assessment was performed to independently assess MV size and thickness. Average leaflet thickness, volume, and total leaflet area were measured using an automated system that independently defines tissue boundaries with a 25 µM^3^ resolution. 

There are both benefits and limitations inherent in any investigation involving murine models of HCM [40,41,42]. In contrast with human HCM, this strain of mice with loss of cMyBP-C does not have LV hypercontractility, SAM, or LVOT obstruction. This model does mimic human disease by targeting the endogenous *Mybpc3* gene, rather than by transgenesis, although (in contrast with patients) hypertrophy is not substantial in the heterozygous mice. Thus, this mouse model is not a perfect model of human HCM, even if histopathological analysis of the LV in these (−/−) mice shows increased LV wall thickness compared to (+/+) mice at 3 months, with associated myofiber disarray and increased interstitial fibrosis, just as in people with HCM [17]. The myocardial walls also produce more CTGF and POSTN, as in other HCM mouse models [23]. Unfortunately, no HCM mouse model with LVOT obstruction is currently available, precluding any comparison between HCM mice with and without obstruction. In about 5% of people with HCM, two mutations (compound/digenic heterozygous or homozygous) are present, typically with a more severe phenotype [43]. There is at least one case published of a person with a homozygous p.Q76Ter nonsense mutation in *MYBPC3* predicted to produce no protein, and this parallels the *Mybpc3* knock-out mouse with a dilated, hypertrophied, and hypokinetic left ventricle and sudden death by 9 months of age [27]. LV enlargement and reduced contractility may be responsible for the increased markers of TGFβ activity, and this may further confound the analysis of MV leaflet length and thickness, despite controlling for these factors.

## 4. Conclusions

*Mybpc3* (−/−) mice with LVH have longer and thicker MV leaflets compared to age-matched control (+/+) mice. Enlargement of the MV leaflets occurs in this model in the absence of SAM and LVOT obstruction. In this murine model of HCM, MV remodeling correlates with multiple markers of increased activity of TGFβ, a pleiotropic family of growth factors that is known to induce MV pathology in other settings, and that has been identified as a critical factor in the pathogenesis of HCM and pathologic MV leaflet elongation. 

## References

[1] Jiang L., Levine R.A., King M.E., Weyman A.E. (1987). An integrated mechanism for systolic anterior motion of the mitral valve in hypertrophic cardiomyopathy based on echocardiographic observations. Am. Heart J..

[2] Hagege A.A., Bruneval P., Levine R.A., Neamatalla H., Judge D.P. (2011). The mitral valve in hypertrophic cardiomyopathy: Old *versus* new concepts. J. Cardiovasc. Transl. Res..

[3] Hagege A.A., Dubourg O., Desnos M., Mirochnik R., Isnard G., Bonne G., Carrier L., Guicheney P., Bouhour J.B., Schwartz K., Komajda M. (1998). Familial hypertrophic cardiomyopathy. Cardiac ultrasonic abnormalities in genetically affected subjects without echocardiographic evidence of left ventricular hypertrophy. Eur. Heart J..

[4] Maron M.S., Olivotto I., Harrigan C., Appelbaum E., Gibson C.M., Lesser J.R., Haas T.S., Udelson J.E., Manning W.J., Maron B.J. (2011). Mitral Valve Abnormalities Identified by Cardiovascular Magnetic Resonance Represent a Primary Phenotypic Expression of Hypertrophic Cardiomyopathy. Circulation.

[5] Schwammenthal E., Levine R.A. (1996). Dynamic subaortic obstruction: A disease of the mitral valve suitable for surgical repair?. J. Am. Coll. Cardiol..

[6] Levine R.A., Vlahakes G.J., Lefebvre X., Guerrero J.L., Cape E.G., Yoganathan A.P., Weyman A.E. (1995). Papillary muscle displacement causes systolic anterior motion of the mitral valve. Experimental validation and insights into the mechanism of subaortic obstruction. Circulation.

[7] Wigle E.D., Adelman A.G., Auger P., Marquis Y. (1969). Mitral regurgitation in muscular subaortic stenosis. Am. J. Cardiol..

[8] Kim D.H., Handschumacher M.D., Levine R.A., Choi Y.S., Kim Y.J., Yun S.C., Song J.M., Kang D.H., Song J.K. (2010). *In vivo* measurement of mitral leaflet surface area and subvalvular geometry in patients with asymmetrical septal hypertrophy: Insights into the mechanism of outflow tract obstruction. Circulation.

[9] Ng C.M., Cheng A., Myers L.A., Martinez-Murillo F., Jie C., Bedja D., Gabrielson K.L., Hausladen J.M., Mecham R.P., Judge D.P. (2004). TGF-β-dependent pathogenesis of mitral valve prolapse in a mouse model of Marfan syndrome. J. Clin. Investig..

[10] Kyndt F., Gueffet J.P., Probst V., Jaafar P., Legendre A., le Bouffant F., Toquet C., Roy E., McGregor L., Lynch S.A. (2007). Mutations in the Gene Encoding Filamin A as a Cause for Familial Cardiac Valvular Dystrophy. Circulation.

[11] Sauls K., de Vlaming A., Harris B.S., Williams K., Wessels A., Levine R.A., Slaugenhaupt S.A., Goodwin R.L., Pavone L., Merot J. (2012). Developmental Basis for Filamin-A Associated Myxomatous Mitral Valve Disease. Cardiovasc. Res..

[12] Disse S., Abergel E., Berrebi A., Houot A.M., le Heuzey J.Y., Diebold B., Guize L., Carpentier A., Corvol P., Jeunemaitre X. (1999). Mapping of a first locus for autosomal dominant myxomatous mitral-valve prolapse to chromosome 16p11.2–p12.1. Am. J. Hum. Genet..

[13] Freed L.A., Acierno J.S., Dai D., Leyne M., Marshall J.E., Nesta F., Levine R.A., Slaugenhaupt S.A. (2003). A locus for autosomal dominant mitral valve prolapse on chromosome 11p15.4. Am. J. Hum. Genet..

[14] Nesta F., Leyne M., Yosefy C., Simpson C., Dai D., Marshall J.E., Hung J., Slaugenhaupt S.A., Levine R.A. (2005). New locus for autosomal dominant mitral valve prolapse on chromosome 13: Clinical insights from genetic studies. Circulation.

[15] Millat G., Bouvagnet P., Chevalier P., Dauphin C., Jouk P.S., da Costa A., Prieur F., Bresson J.L., Faivre L., Eicher J.C. (2010). Prevalence and spectrum of mutations in a cohort of 192 unrelated patients with hypertrophic cardiomyopathy. Eur J. Med. Genet..

[16] Watkins H., Conner D., Thierfelder L., Jarcho J.A., MacRae C., McKenna W.J., Maron B.J., Seidman J.G., Seidman C.E. (1995). Mutations in the cardiac myosin binding protein-C gene on chromosome 11 cause familial hypertrophic cardiomyopathy. Nat. Genet..

[17] Carrier L., Knoll R., Vignier N., Keller D.I., Bausero P., Prudhon B., Isnard R., Ambroisine M.L., Fiszman M., Ross J. (2004). Asymmetric septal hypertrophy in heterozygous cMyBP-C null mice. Cardiovasc. Res..

[18] Lekanne Deprez R.H., Muurling-Vlietman J.J., Hruda J., Baars M.J.H., Wijnaendts L.C.D., Stolte-Dijkstra I., Alders M., van Hagen J.M. (2006). Two cases of severe neonatal hypertrophic cardiomyopathy caused by compound heterozygous mutations in the MYBPC3 gene. J. Med. Genet..

[19] Xin B., Puffenberger E., Tumbush J., Bockoven J.R., Wang H. (2007). Homozygosity for a novel splice site mutation in the cardiac myosin-binding protein C gene causes severe neonatal hypertrophic cardiomyopathy. Am. J. Med. Genet. A.

[20] Bhan A., Sirker A., Zhang J., Protti A., Catibog N., Driver W., Botnar R., Monaghan M.J., Shah A.M. (2014). High-frequency speckle tracking echocardiography in the assessment of left ventricular function and remodeling after murine myocardial infarction. Am. J. Physiol. Heart Circ. Physiol..

[21] Henning A.L., Jiang M.X., Yalcin H.C., Butcher J.T. (2011). Quantitative three-dimensional imaging of live avian embryonic morphogenesis via micro-computed tomography. Dev. Dyn..

[22] Norris R.A., Potts J.D., Yost M.J., Junor L., Brooks T., Tan H., Hoffman S., Hart M.M., Kern M.J., Damon B. (2009). Periostin promotes a fibroblastic lineage pathway in atrioventricular valve progenitor cells. Dev. Dyn..

[23] Teekakirikul P., Eminaga S., Toka O., Alcalai R., Wang L., Wakimoto H., Nayor M., Konno T., Gorham J.M., Wolf C.M. (2010). Cardiac fibrosis in mice with hypertrophic cardiomyopathy is mediated by non-myocyte proliferation and requires TGF-β. J. Clin. Investig..

[24] Massague J., Seoane J., Wotton D. (2005). Smad transcription factors. Genes Dev..

[25] Habashi J.P., Judge D.P., Holm T.M., Cohn R.D., Loeys B.L., Cooper T.K., Myers L., Klein E.C., Liu G., Calvi C. (2006). Losartan, an AT1 Antagonist, Prevents Aortic Aneurysm in a Mouse Model of Marfan Syndrome. Science.

[26] Morner S., Richard P., Kazzam E., Hellman U., Hainque B., Schwartz K., Waldenstrom A. (2003). Identification of the genotypes causing hypertrophic cardiomyopathy in northern Sweden. J. Mol. Cell. Cardiol..

[27] Richard P., Charron P., Carrier L., Ledeuil C., Cheav T., Pichereau C., Benaiche A., Isnard R., Dubourg O., Burban M. (2003). Hypertrophic cardiomyopathy: Distribution of disease genes, spectrum of mutations, and implications for a molecular diagnosis strategy. Circulation.

[28] Klues H.G., Maron B.J., Dollar A.L., Roberts W.C. (1992). Diversity of structural mitral valve alterations in hypertrophic cardiomyopathy. Circulation.

[29] Kaple R.K., Murphy R.T., DiPaola L.M., Houghtaling P.L., Lever H.M., Lytle B.W., Blackstone E.H., Smedira N.G. (2008). Mitral valve abnormalities in hypertrophic cardiomyopathy: Echocardiographic features and surgical outcomes. Ann. Thorac. Surg..

[30] Mautner S.L., Klues H.G., Mautner G.C., Proschan M.A., Roberts W.C., Maron B.J. (1993). Comparison of mitral valve dimensions in adults with valvular aortic stenosis, pure aortic regurgitation and hypertrophic cardiomyopathy. Am. J. Cardiol.

[31] Shah P.M., Taylor R.D., Wong M. (1981). Abnormal mitral valve coaptation in hypertrophic obstructive cardiomyopathy: Proposed role in systolic anterior motion of mitral valve. Am. J. Cardiol..

[32] Schwammenthal E., Nakatani S., He S., Hopmeyer J., Sagie A., Weyman A.E., Lever H.M., Yoganathan A.P., Thomas J.D., Levine R.A. (1998). Mechanism of Mitral Regurgitation in Hypertrophic Cardiomyopathy: Mismatch of Posterior to Anterior Leaflet Length and Mobility. Circulation.

[33] Petrone R.K., Klues H.G., Panza J.A., Peterson E.E., Maron B.J. (1992). Coexistence of mitral valve prolapse in a consecutive group of 528 patients with hypertrophic cardiomyopathy assessed with echocardiography. J. Am. Coll. Cardiol..

[34] Chaput M., Handschumacher M.D., Tournoux F., Hua L., Guerrero J.L., Vlahakes G.J., Levine R.A. (2008). Mitral leaflet adaptation to ventricular remodeling: Occurrence and adequacy in patients with functional mitral regurgitation. Circulation.

[35] Messas E., Bel A., Szymanski C., Cohen I., Touchot B., Handschumacher M.D., Desnos M., Carpentier A., Menasche P., Hagege A.A. (2009). Relief of mitral leaflet tethering following chronic myocardial infarction by chordal cutting diminishes left ventricular remodeling. Circ. Cardiovasc. Imaging.

[36] Judge D.P., Biery N.J., Keene D.R., Geubtner J., Myers L., Huso D.L., Sakai L.Y., Dietz H.C. (2004). Evidence for a critical contribution of haploinsufficiency in the complex pathogenesis of Marfan syndrome. J. Clin. Investig..

[37] Judge D.P., Rouf R., Habashi J., Dietz H.C. (2011). Mitral valve disease in Marfan syndrome and related disorders. J. Cardiovasc. Transl. Res..

[38] Gould R.A., Sinha R., Aziz H., Rouf R., Dietz H.C., Judge D.P., Butcher J. (2012). Multi-scale biomechanical remodeling in aging and genetic mutant murine mitral valve leaflets: Insights into Marfan syndrome. PLoS One.

[39] Seidman J.G., Seidman C. (2001). The Genetic Basis for Cardiomyopathy: From Mutation Identification to Mechanistic Paradigms. Cell.

[40] Harris S.P., Lyons R.G., Bezold K.L. (2011). In the Thick of It. Circ. Res..

[41] Shephard R., Semsarian C. (2009). Role of animal models in HCM research. J. Cardiovasc. Transl. Res..

[42] McConnell B.K., Jones K.A., Fatkin D., Arroyo L.H., Lee R.T., Aristizabal O., Turnbull D.H., Georgakopoulos D., Kass D., Bond M. (1999). Dilated cardiomyopathy in homozygous myosin-binding protein-C mutant mice. J. Clin. Investig..

[43] Judge D.P., Johnson N.M. (2008). Genetic evaluation of familial cardiomyopathy. J. Cardiovasc. Transl. Res..

